# Are Future Psychologists Willing to Accept and Use a Humanoid Robot in Their Practice? Italian and English Students’ Perspective

**DOI:** 10.3389/fpsyg.2019.02138

**Published:** 2019-09-18

**Authors:** Daniela Conti, Allegra Cattani, Santo Di Nuovo, Alessandro Di Nuovo

**Affiliations:** ^1^Sheffield Robotics, Faculty of Arts, Computing, Engineering and Sciences, Sheffield Hallam University, Sheffield, United Kingdom; ^2^School of Psychology, University of Plymouth, Plymouth, United Kingdom; ^3^Department of Educational Sciences, University of Catania, Catania, Italy

**Keywords:** socially assistive robotics, cross-cultural analysis, humanoid robot, psychological practice, UTAUT model

## Abstract

Despite general scepticism from care professionals, social robotics research is providing evidence of successful application in education and rehabilitation in clinical psychology practice. In this article, we investigate the cultural influences of English and Italian psychology students in the perception of usefulness and intention to use a robot as an instrument for future clinical practice and, secondly, the modality of presentation of the robot by comparing oral vs. video presentation. To this end, we surveyed 158 Italian and British-English psychology students after an interactive demonstration using a humanoid robot to evaluate the social robot’s acceptance and use. The Italians were positive, while the English were negative toward the perceived usefulness and intention to use the robot in psychological practice in the near future. However, most English and Italian respondents felt they did not have the necessary abilities to make good use of the robot. We concluded that it is necessary to provide psychology students with further knowledge and practical skills regarding social robotics, which could facilitate the adoption and use of this technology in clinical settings.

## Introduction

The Socially Assistive Robot (SAR) is a fast emerging technology that has developed from the cross-over of social robotics and assistive robotics and involves robots that are designed to support people’s everyday lives through advanced social interaction (Feil-Seifer and Matarić, 2009). Such robots are designed to help people through advanced interaction driven by user needs (e.g., companionship, physical therapy, daily living assistance, tutoring) via multimodal interfaces (speech, graphical gestures, and input devices) (Feil-Seifer and Matarić, 2011). Therefore, these socially capable robots can assist in everyday human activities ranging from tutoring an elderly person in physical exercise, assisting either teachers in telling pre-recorded tales to pre-school children (Conti et al., 2019), in teaching a second language (Alemi et al., 2014), guiding visitors in museums (Yousuf et al., 2012; Fasola and Matarić, 2013), and supporting parents in home education (Han et al., 2005).

Given the increased interest toward education and care for people, in recent years there has been a large amount of work involving the acceptability of robots as mediators between elderly assistance and home technology (De Ruyter and Aarts, 2004; Mayer and Panek, 2014), capable of providing assistance to diabetics (Looije et al., 2006), performing the role of teaching assistant (You et al., 2006), carrying out studies of behaviour proactive, measuring blood pressure (Kuo et al., 2009), and motivating users to perform physical activities (Klamer and Ben Allouch, 2010; Heerink, 2011).

Despite successful scientific experimentation and increasing positive evidence and applications, it seems that most people are still sceptical or actually against the use of robots in real contexts. In a recent European survey (European Commission, 2017), only 26% of respondents were comfortable “*with having a robot to provide services and companionship when infirm or elderly*” or “*with having a medical operation performed on them by a robot*.”

One of the areas in which this discrepancy is more relevant is the area of psychological practice (Diehl et al., 2012) in children and elderly people. SAR research has successfully demonstrated the therapeutic benefits of such use in clinical and health psychology of children with developmental disabilities, and those with autism spectrum disorder (Robins et al., 2005; Kim et al., 2012; Scassellati et al., 2012; Conti et al., 2018; Richardson, 2018), and to improve the social skills during a long-term home-based intervention (Scassellati et al., 2018). Recently, Taheri et al. (2019) reported the scepticism of therapists about the use of an assistant robot with the aid of the music teaching. The results of a recent European project (Cao et al., 2019) showed that the *Robot-Enhanced Therapy* was a promising approach that could be as efficient as classical interventions for a large variety of outcomes for children with ASDs. However, the authors concluded that few participants could benefit from the system developed in the project (Cao et al., 2019).

Socially Assistive Robot application was also successfully demonstrated in helping the health education of children with diabetes (Looije et al., 2006; Blanson Henkemans et al., 2013). Likewise, it has helped elderly people suffering from a variety of neurological and psychiatric conditions (Rabbitt et al., 2015). This evidences has led scholars to recommend a stable integration of Human-Robot Interaction (HRI) in psychological healthcare (Iroju et al., 2017). Although, in previous work, we reported that Italian experienced practitioners showed an overall positive attitude toward the use of such robots, practitioners are still sceptical and perceive the assistive robot as an expensive and limited tool (Conti et al., 2017c). Scassellati et al. (2012) have suggested that this could be due to the limited involvement of actual practitioners in the development of SAR applications.

To further analyse the psychologists’ opinion and develop better suited applications in this work, we extended the previous research (Conti et al., 2017c) by focusing on psychology students and performing a cross-cultural analysis on the perception of a humanoid robot as an instrument for their future practice. We valued the students’ opinion because they were more likely to be the real beneficiaries of the current research, i.e., they will be psychology practitioners when current prototypes become commercially available and certified for clinical use.

## Background and Hypotheses

### Presentation Modality of Robot Capabilities

The reasons for the discrepancy between the benefits showed by research and people’s perception and attitude toward robots has been debated (e.g., Moon et al., 2012; Coeckelbergh et al., 2015). However, the European survey (European Commission, 2017) also reports that a large majority of respondents (85%) have never used a real robot, and only 47% have “*heard, read or seen something about artificial intelligence in the last 12 months*.” But those who had recently learned about robotics were “*more likely to have a positive view*” (European Commission, 2017). Therefore, we hypothesize that people are anxious toward the social use of robots because they are mostly unfamiliar with the concept.

For this reason, we decided to focus on the way robots are presented, as this might partially explain the difference of perception between researchers and public opinion. To date, the expectation of SARs has been shaped particularly by mainstream media (e.g., books, internet, and movies) mostly with fictional scenarios and not live interactions with physically present robots (Haring et al., 2014). Some studies evidenced how previous exposure through either media or personal interactive experiences with robots could play an important role in determining possible differences in attitude (Broadbent et al., 2009). For example, Goetz et al. (2003) and Lohse et al. (2008) presented pictures or videos of robots but no real interaction between the participants and the robots occurred. The participants reported quite diverse ideas on robots’ application and each application was described within a certain context. The authors stressed that people’s perception of appropriate tasks for a robot might be different if the robot were to interact with them in the real world. However, Xu et al. (2015) investigated the influence of showing a video in an online review on consumer perceptions and decisions to purchase products and demonstrated that video as a format has a significant positive influence on consumer perceptions and their intention to purchase, and increases the perception of helpfulness/usefulness.

Furthermore, it has been shown in other studies that the physical presence of a robot during an interaction plays a significantly positive role in how people perceive the robot (Bainbridge et al., 2011; Krogsager et al., 2014). Bainbridge et al. (2011) examined three conditions separately: *physical*, *live-video*, and *augmented-video*. In the physical condition participants performed the task in the same room as the robot. In the live-video condition, participants interacted with a live video feed of the robot displayed on a monitor. In the augmented-video condition, participants interacted with two monitors, one with live-video condition, and one with an overhead video of the robot. The researchers concluded that participants rated the interaction with the physically present robot more positively and as more natural than with the video-displayed robot, suggesting better human interactions occur with a physically present robot (Bainbridge et al., 2011). In another study, the authors considered the use of head nods in communication and compared the use of virtual agents and a physically present humanoid robot (NAO), concluding that the physical robot had more impact on the user than the virtual agent (Krogsager et al., 2014).

We decided to include a video along with the physical experience to verify the effect of a standardized presentation of the possible applications and to evaluate the impact of this multimedia experience on their perceptions of the robot.

For this reason, we tested the following hypothesis:

*H1*: An additional video presentation has a positive influence on Perceived Usefulness (PU) and Intention to Use (ITU) the robot.

### Cultural Background

Culture refers to a set of shared norms, values, and patterns of orientation that influence the behaviour of individuals within groups or collectives like nations, organizations, and teams (Hofstede and Hofstede, 2001; Straub et al., 2002).

Cross-cultural differences have been investigated across many disciplines and the field of HRI is no exception. It is therefore believed that cultural background may also influence the way people perceive robots.

The results should be useful in designing service robots that will be operating across cultures. Recent HRI research has investigated in particular how cultural backgrounds affect people’s reaction to and perception of a robot, and to determine the proper appearance for specific tasks in a cross-cultural context (Li et al., 2010; Shahid et al., 2014; Haring et al., 2016). The work that investigated the cultural differences in acceptance of robotic services, focussed on western vs. eastern backgrounds as there is a stereotype that Asian (e.g., Confucian, Southeast Asian) people perceive robots more positively than people from Western backgrounds (e.g., Anglo, Nordic and Germanic). However, this is debated by the scientific community: some authors indicate that this is not necessarily true (MacDorman et al., 2009; Haring et al., 2014, 2016), while others report that Asian and Western people perceive robots differently with regard to animacy, intelligence, and safety (Haring et al., 2015).

Very few researchers have studied the differences between Italian and British cultures, who are often grouped without distinction in the Western block, even if they belong to two different cultural clusters, Latin Europe and Anglo, as recently underlined by the GLOBE study (House et al., 2004).

Moreover, the Italian and United Kingdom education systems are different, with the overall Italian education system, being theoretically oriented and teacher-centred with the didactic method of teaching (Cangelosi, 2012), and with the students being required to build a portfolio of practical skills later, just before or after graduation; while in the United Kingdom system the teaching is focused from the beginning on practical sessions and experiential learning where students are expected to acquire the necessary skills to have direct access to skilled employment in the United Kingdom (Light et al., 2009; Land, 2010).

Therefore, for these reasons, we tested the following hypothesis:

*H2*: Perceived Usefulness (PU) and Intention To Use (ITU) a robot in one’s future profession are different between English and Italian psychology students.

## Materials and Methods

### Participants

Our experimental sessions attracted 158 MPsych students consisting of Italian students (*n* = 80, Males = 6, Females = 74, M-age = 25.1 years, range = 22–30, SD = 2.17) recruited at the University of Catania and the British-English students (*n* = 78, Males = 16, Females = 62, M-age = 20.6 years, range = 19–30, SD = 2.21) recruited at the University of Plymouth. Gender imbalance reflects the population ratio in most psychology degree courses equivalent to over 80% of women.

The English students were awarded one learning credit for the attendance. All the participants we included had no previous experience of interaction with social robotic platforms, nor had the use of robots been previously presented to them as an instrument for their professional practice.

Both the English and Italian students were invited to take part in one of eight group sessions (4 + 4) involving the around 20 participants, and they randomly attended either an oral (*n* = 92: Italy = 57, United Kingdom = 35) or oral and video presentation (*n* = 66: Italy = 23, United Kingdom = 43). Attendance at these sessions was uneven with some groups larger than others because participation was voluntary. Students didn’t know in advance which modality of presentation was going to be delivered.

Ethical approval was obtained by the relevant University boards in Plymouth and Catania. Informed consent to participate and to use data for scientific research was obtained from all participants prior to the study. The methods were carried out in accordance with the relevant guidelines and regulations for human subjects.

### Questionnaire

In this study, we used and adapted the questionnaire proposed in Heerink et al. (2009), which has been widely used in SAR research and has been found to be highly reliable in several previous studies (among others: De Ruyter et al., 2005; Looije et al., 2006; Heerink et al., 2010; de Graaf and Ben Allouch, 2013; Fridin and Belokopytov, 2014; Conti et al., 2015a, 2017a; Di Nuovo et al., 2018a). The questionnaire is based on the Unified Theory of Acceptance and Use of Technology (UTAUT) developed by Venkatesh et al. (2003). The original UTAUT model (Venkatesh et al., 2003) has been cross-culturally tested in the Czechia, Greece, India, Malaysia, New Zealand, Saudi Arabia, South Africa, United Kingdom, and United States students (Li et al., 2010). The authors (Li et al., 2010) show that the UTAUT part of the questionnaire used by them can measure the influence of national culture. Based on this, they concluded that the UTAUT model is robust and applicable across diverse countries and cultures.

The constructs represented by a few questions and the scores for the constructs can be mapped and interrelated. In particular:

• ANX – Anxiety in the perception of robots: evoking anxious or emotional reactions when using the robot;
• ATT – Attitude to use them: positive or negative feelings about the appliance of the technology;
• FC – Facilitating Conditions in their use: objective factors in the environment that facilitate using the robot;
• ITU – Intention To Use: the outspoken intention to use the robot over a longer period of time;
• PAD – Perceived Adaptability: the perceived ability of the robot to be adaptive to the changing needs of the user;
• PENJ – Perceived Enjoyment: feelings of joy or pleasure associated by the user with the use of the robot;
• PS – Perception of Sociability: the perceived ability of the robot to perform sociable behaviour;
• PU – Perceived Usefulness: the degree to which a person believes that using the system would enhance his or her daily activities;
• SI – Social Influence: the user’s perception of what people who are important to him think about him using the robot;
• SP – Social Presence: the experience of sensing a social entity when interacting with the robot;
• TRU – Trust: the belief that the robot can perform with personal integrity and reliability.

For the purpose of this work, we modified question 6, where the word “life” was replaced with “future job” in both language versions, and we did not refer to the *iCat* robot (as this was the robotic platform tested in the original questionnaire). In order to make a cross-cultural comparison, we translated the original English UTAUT questionnaire (Heerink et al., 2009) into Italian and then again back into English to ensure translation equivalence (Brislin, 1970). The adapted version was given to the Italian students. We piloted the questionnaire with four English and Italian University students to confirm the clarity of the instructions, the wording of the questions, and to receive any comments to identify potential issues. These versions were previously used in preliminary work (Conti et al., 2015a), where they showed the potential to discriminate between Italian and English students.

The questionnaire items are listed in the Appendix.

### The NAO Humanoid Robotic Platform

The robotic platform used is a 58 cm tall NAO humanoid robot (Gouaillier et al., 2009) weighing 4.3 kg provided by the robot manufacturer Softbank Robotics. The NAO robot looks like a toy and has 25 degrees of freedom (four joints for each arm; two for each hand; five for each leg; two for the head and one to control the hips), which allows it to perform a variety of movements. This robot can detect faces and respond to eye contact by moving its head accordingly. It can also vary the colour of LEDs in its eyes’ contour to simulate emotions, and it can capture a lot of information about the environment using sensors and microphones. The NAO robot has pioneered the use of robotic toys as therapeutic and educational aides and is widely used in SAR (Shamsuddin et al., 2012; Kim et al., 2013; Di Nuovo et al., 2018b), particularly in acceptance studies (e.g., de Graaf and Ben Allouch, 2013; Kim et al., 2013). To program the NAO’s behaviours we used *Choregraphe*, a development environment provided by the robot manufacturer (Pot et al., 2009). Using *Choregraphe*, we developed a set of pre-programmed behaviours to allow the robot to interact with the participants and we installed them on the NAO’s memory.

## Experimental Procedure

The experiment consisted of two sessions: the interactive session and the questionnaire session. Each session lasted about 30 min. The experimental procedure was the same for all participants, apart from the Presentation phase. Details of experimental procedure are in the Supplementary Material.

### Interactive Session

The lecture rooms had good light and there was no noise. The students could move freely into the room to watch a NAO robot sitting on a table or moving on the floor. Two researchers were present in the room. One researcher was always close to the robot, explaining its features and starting the activities. The other researcher was checking the robot’s sensors (e.g., cameras, battery) to verify all parameters and act in case of technical problems. The interactive session consisted of three phases.

#### Warming Up Phase

The NAO robot was placed in the middle of a table with good visibility to all participants.

#### Presentation Phase

One of the researchers briefly explained the scope of the research and presented the NAO robot. Then this was followed by either:

• An oral presentation: this consisted of around 6 min of oral presentation, introducing the robot, the way of programming it and giving examples of recent research in the psychological profession (the same of the video presentation below). The presenter was always the same person for both groups, he was an experienced researcher in technology applications to psychology, and he had an extensive track record of oral presentations and teaching to university students in both Italy and the United Kingdom.
• A video presentation, i.e., a 6-min video showing real examples of possible applications of the NAO robot in various contexts, such as in a school with children (Conti et al., 2017b, 2019), in the therapeutic treatment of children with intellectual disabilities (Conti et al., 2015b), and in hospital with children (Beran et al., 2013) and the elderly (Sarabia et al., 2018). To avoid language barriers, the video didn’t have any audio, but subtitles in the local language.

The contents of the oral presentation were aligned to the content of the video presentation to give the same information to all students. The presenter strictly followed a script that was created via the back-translation method. Both presentations were honestly reporting strengths and weaknesses of the robot.

#### NAO Interactive Live and Game Phase

**FIGURE 1:**
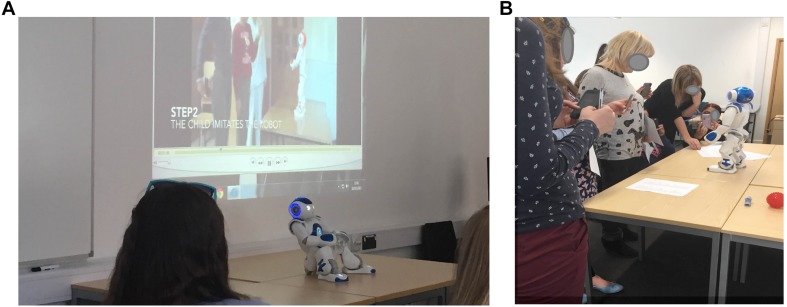
Students watching the video on the left **(A)**, NAO robot playing with students on the right **(B)**.

All participants took part. The NAO robot was turned on to perform welcoming and greeting actions plus five activities. After the NAO robot welcomed the participants, it danced to show the harmony of its movements. For the first activity, the NAO robot was turned to the “autonomous life mode,” which consisted of the students asking questions which the robot had to answer. Students had a list of the possible questions and, in turn, spoke to the robot, testing its speech recognition abilities. Topics were varied: questions relating to the robot, such as gender, mood, skills recognition, weather, etc. Next, the robot proposed an interactive game of image recognition. Sheets with printed images were placed on the table that a random volunteer selected and showed to the robot when asked questions such as “Show me a tree” or “I would like to see a star.” The game was repeated several times to allow as many participants as possible to personally interact with the robot. This part of the experiment is represented in Figure 1. The third activity was to demonstrate the ability of exploration mobility; the NAO identified and followed a red ball that was waved in front of it by volunteers. In the fourth activity the NAO asked to place in its hand an object. It then grasped the object, but returned it saying that the object was not interesting. The last activity was an exploration game where the robot was placed on the floor in a walking mode for 5 min. The robot then walked around the room among the participants responding to commands given by the students regarding the direction (forward, backward, turn left and right) in which the robot should move.

The activities were selected to be representative of the ones typically used in the clinical and educational psychology applications, especially of those included in presentations.

All observers were at the same distance from the robot during the interaction session and demonstrated active involvement during the interactive game. Finally, the robot thanked the participants for attending and for their participation. During the Interactive live and game phase, students could ask for clarifications to the researchers, but only about the interaction with the NAO robot and the applications presented to avoid differences between groups. This to reduce the possibility of misunderstandings and made more homogenous the perception by the students.

### Questionnaire Session

In the second part of the experiment, participants were asked to fill in the questionnaire and to rate the extent to which they agreed/disagreed with the statements in the questionnaire as future psychologist. The questionnaire was anonymous, apart from some generic details such as gender, age, and nationality. The participants indicated their level of agreement with 36 statements grouped in 11 constructs on five-point Likert scales including verbal anchors: “*totally disagree*” (1) – “*disagree*” (2) – “*neither agree nor disagree*” (3) – “*agree*” (4) – “*totally agree*” (5). At the end, a final discussion was held to allow students to express their own thoughts and to debrief the students about the procedure and purpose of the research.

## Data Analyses and Results

### Data Analyses

For each participant we calculated the average scores of the items that constituted each of the eleven constructs of the UTAUT questionnaire, e.g., ANX score is the average of the ANX1, ANX2, ANX3, and ANX4 scores.

The statistical analyses included a Multivariate General Linear Model to compare the means of the eleven constructs with nationality (English vs. Italian) and the Presentation type (oral vs. video) between subject factors to test the impact of culture and the mode of presentation.

We performed the *t*-test comparisons on the scores of the constructs of the questionnaire for equality of group means of average scores and the percentage of participants with a positive or negative perception. A positive agreeability perception (POS) of a participant for a given construct is assumed when the average score is greater than 3, while a negative disagreeability perception (NEG) is given when the average score is less than 3. Otherwise, the perception is considered neutral.

Further, we tested the capability of UTAUT constructs and questionnaire components to reveal the country of the participants via the generation of a decision tree classifier from the constructs and a stepwise discriminant analysis of the single items. These analyses were applied to reduce the variables and identify those constructs or statements of the questionnaire that could be used to predict the country of study and, therefore, to highlight the differences between the two cultures.

The decision tree classifier is used here to discover and visualize the relationships among the questionnaire’s constructs and the profile of the cultural samples. A decision tree is “*a representation of a decision procedure for determining the class of a given instance*” (Utgoff, 1989). Each node of the tree can identify either a class name or a specific test that can further partition the space according to a small set of possible results of the test. Each subset of the partition corresponds to a classification sub-problem for that subspace of the instances, which is solved by a subtree. A decision tree can be seen as a *divide-and-conquer* strategy for object classification. In practice, one can define a decision tree to be either (Utgoff, 1989): *a “leaf node”* (or “*answer node”*), which contains a class name, or *a “non-leaf node”* (or “*decision node”*), which contains an attribute test with a branch to another decision tree for each possible value of the attribute. For more details and a comprehensive discussion of decision trees and their variants (see Safavian and Landgrebe, 1991; Murthy, 1998).

The stepwise discriminant analysis is used to find the most relevant questionnaire statements and define a model that relates these to the country of study. We performed this analysis with the aim of identifying numerical relations among the statements scores, which can be used to find general characteristics of the two cultural samples.

The algorithm selected for creating the decision tree is the Chi-squared Automatic Interaction Detector (CHAID) (Kass, 1980), a fast, multi-way tree algorithm that explores data quickly and efficiently, and builds segments and profiles for categorical variables. In our experiment, we set the minimum size of a node to 10 to maximize readability and performance; all the remaining parameters are the default of the SPSS package.

In building the model with the discriminant analysis, the criterion used for controlling the stepwise entry of variables was the Wilks’ lambda. The Wilks’ lambda criterion is a measure of group discrimination. Variables for entry into the equation were chosen based on how much they lower Wilks’ lambda. At each step, the variable that minimizes the overall Wilks’ lambda is entered.

A cross-validation is also performed with the leave-one-out method: each case in the analysis is classified using the function derived from the other remaining cases.

The decision tree was created using the constructs’ scores, while the discriminant analysis was done using either the constructs’ scores or the raw scores from the 36 items in the questionnaire.

All statistics were calculated using SPSS 24 software. In our statistical analyses, we used default parameters unless otherwise specified. In the case of decision tree analysis, statistically significant results are when *p* < 0.001, while for the other analyses they are *p* < 0.01.

### Results

In Table 1 the percentage of positive and negative scores shows the country differences in the ITU construct, where most Italian respondents (63%) gave positive scores while for the United Kingdom respondents the negative scores form the majority (59%). We note that in the case of Trust construct both cultures scored negatively. The students demonstrated that they enjoyed interacting with the robot (overall M-PENJ = 4.20, SD = 0.67), and felt little anxiety interacting with it (overall M-ANX = 4.38, SD = 0.81). They had also positive feelings about having a robot in their learning environment (overall M-ATT = 3.88, SD = 0.76).

**TABLE 1:** Descriptive statistics, mean with standard deviations, result of the *t*-test, and percentages of positive (>3; agree) vs. negative (<3; disagree) perception for each construct of Italian and English students.

	**Italian**						**English**						**Mean**	***t***	***p***
					
	**Mean**	**Min**	**Max**	**SD**	**POS (%)**	**NEG (%)**	**Mean**	**Min**	**Max**	**SD**	**POS (%)**	**NEG (%)**	**Diff.**		
ANX	4.39	3.00	5.00	0.65	91	0	4.37	1.00	5.00	0.95	87	12	0.02	0.169	0.866
ATT	3.88	2.00	5.00	0.68	83	4	3.88	1.00	5.00	0.84	85	6	0.00	−0.009	0.993
FC	2.33	1.00	4.00	0.85	15	71	2.50	1.00	4.50	0.89	17	63	−0.18	−1.264	0.208
ITU	3.34	1.00	5.00	0.69	63	10	2.55	1.00	5.00	1.12	32	59	**0.79**	**5.351**	**<0.001**
PAD	3.38	1.00	5.00	0.88	66	15	3.35	1.00	5.00	0.83	64	23	0.29	0.212	0.833
PENJ	4.20	3.00	5.00	0.52	99	0	4.20	1.60	5.00	0.80	91	6	0.00	−0.023	0.982
PS	3.32	1.50	5.00	0.67	63	16	3.39	1.50	4.80	0.76	60	24	−0.07	−0.608	0.544
PU	3.46	1.30	4.70	0.61	73	6	3.00	1.00	5.00	1.02	49	47	**0.45**	**3.402**	**0.001**
SI	3.38	1.00	5.00	0.74	59	15	3.17	1.00	5.00	0.99	50	26	0.21	1.502	0.135
SP	2.52	1.00	4.20	0.74	24	64	2.73	1.00	4.80	0.87	33	59	−0.21	−1.613	0.109
TRU	2.18	1.00	5.00	1.06	11	61	2.64	1.00	5.00	1.11	29	55	**−0.46**	−**2.660**	**0.009**

*Statistically significant (*p* < 0.01) difference are shown in bold.*

We tested the impact of the video presentation on the UTAUT questionnaire’s constructs. The Multivariate GLM reported a main significant effect of Country (Italy vs. United Kingdom), *F*(11,144) = 7.47, *p* < 0.01; Wilk’s Λ = 0.637, η*_p_*^2^ = 0.363. The between-subject effects of Country revealed three significant constructs: Intention to use (ITU) *F*(1,154) = 31.18, *p* < 0.001; η*_p_*^2^ = 0.168; Perceived Usefulness (PU) *F*(1,154) = 12.26, *p* = 0.001; η*_p_*^2^ = 0.074, and Trust (TRU) *F*(1,154) = 5.05, *p* = 0.026; η*_p_*^2^ = 0.032. Therefore, the Italian students were more willing to use the robot (Italy M-ITU = 3.34, SE = 0.11; United Kingdom M-ITU = 2.55, SE = 0.11), and had a higher perceived usefulness in their future profession than the English students (Italy M-PU = 3.46, SE = 0.100; United Kingdom M-PU = 3.00, SE = 0.100), but on the other hand the Italian students were less likely to follow advice from the robot than the English students (Italy M-TRU = 2.18, SE = 0.140; United Kingdom M-TRU = 2.64, SE = 0.120).

The main effect of Presentation type (oral vs. video) was not significant, *F*(11,144) = 1.41, *p* = 0.173; Wilk’s Λ = 0.903, η*_p_*^2^ = 0.097. Hence, the type of presentation (oral vs. video) before the live demonstration made no difference in the students’ perception. Also, the Country x Video interaction, *F*(11,144) = 0.190, *p* = 0.998; Wilk’s Λ = 0.985, η*_p_*^2^ = 0.015 was not significant.

Statistically significant (*p* < 0.01) differences are highlighted in bold in Table 1. Positive values for *t* and difference identify a higher mean score of the Italy sub-sample; conversely a negative value identifies a higher mean of United Kingdom students.

**FIGURE 2:**
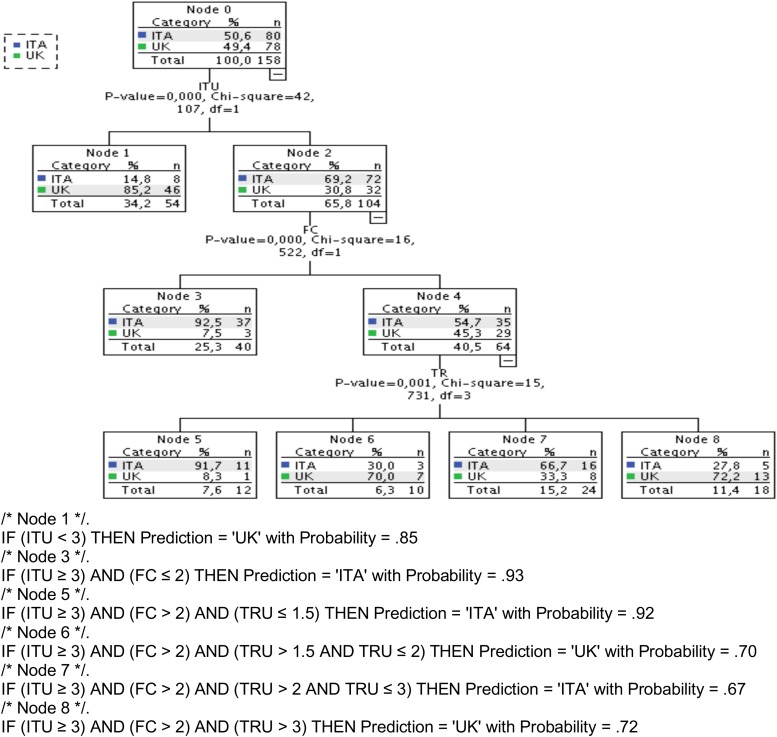
Decision Tree diagram and classification rules for final nodes.

Using the CHAID method, the best possible decision tree for classifying the participants into the two sub-samples has 20 nodes with an overall classification performance of 85.4% (Italy 88.8%, United Kingdom 82.1%, risk of estimate = 0.146, SE = 0.028) demonstrating how the questionnaire scores can predict the country of study and its capability to highlight the cultural differences. However, a tree with 20 nodes doesn’t make a meaningful graphical inspection of the relationships among constructs possible, which is the main feature of the decision trees. For this reason, Figure 2 (top) presents the diagram of a smaller tree composed of eight nodes, which has been obtained applying stricter criterion for splitting the nodes (*p* < 0.001) than the default. The classification capability is still noteworthy with an overall performance of 82.3% correctly classified participants (Italy 80.0%, United Kingdom 84.6%, risk of estimate = 0.177, SE = 0.030).

The bottom part of Figure 2 presents the classification rules of the final nodes with the probability of the prediction. It should be noted that while the ITU construct alone is able to separate quite effectively the two countries of study, other variables (FC and TRU) are needed to refine the classification.

After a visual inspection of the decision tree nodes, we can see that the majority of United Kingdom students (*N* = 46; 59%) can be identified by the negative score to ITU (<3), while most Italian students (*N* = 37, 46%; node 3, top Figure 2) score the facilitating conditions negatively (FC ≤ 2) but they don’t exclude the possibility to use the robot (ITU ≥ 3). Within the group of participants with a positive ITU, we can also distinguish a large sub-group of Italian students (*N* = 27, 34%; nodes 5 and 7) that are characterized by a negative trust (TRU ≤ 3) but are less negative about the conditions to make good use of the robot (FC > 2), and a sub-group of United Kingdom students (*N* = 13, 17%; node 8) that have a positive ITU and trust (TRU > 3).

The correlation analysis identified 14 items among the UTAUT questionnaire statements that exhibit significant (*p* < 0.05) relations to the participant country of study: ANX-2, ATT-5, ATT-6, ITU-10, ITU-11, ITU-12, PS-21, PS-24, PU-25, PU-26, PU-27, SP-31, TR-35, and TRU-36. From these 14 items, the stepwise discriminant analysis identified six questionnaire statements (for a list of the statements, see Table 2), which produced a very high degree of separation between the two country groups (Italy and United Kingdom) as indicated by the final Wilks’ lambda (Λ = 0.533, χ^2^ = 96.31, *p* < 0.001), and the canonical correlation (*r* = 0.683, Eigenvalue = 0.877) for the discriminant function identified.

**TABLE 2:** Standardized canonical discriminant function coefficients.

**Item**	**Statement**	**Coefficient**
ITU-12	“*I’m planning to use the robot in the near future*”	1.057
SP-32	“*I can imagine the robot to be a living creature*”	0.360
PU-25	“*I think the robot is useful to me*”	0.337
PENJ-19	“*I find the robot fascinating*”	−0.316
FC-8	“*I have everything I need to make good use of the robot*”	−0.501
ATT-6	“*The robot would make my life more interesting*”	−0.890

The canonical discriminant function variables and coefficients are reported in Table 2. Two of the items identified are part of the significantly different constructs (ITU-12, PU-25), while the other contribution is from attitude (ATT-6), facilitating conditions (FC-8) and attributes related to social perception (PENJ-19 and SP-32). Positive coefficients of the discriminant function indicate that higher scores for that statement push the classification toward the Italian, while negative coefficients relate higher scores to the English students.

The classification results presented in Table 3 confirm that the discriminant function can effectively distinguish two different groups, Italy and United Kingdom. In fact, 87.3% participants are correctly classified using the whole sample to derive the discriminant function; while with cross-validation 84.8% of cases are classified correctly, confirming the very good predictive value of the questionnaire.

**TABLE 3:** Discriminant analysis: classification results using the entire sample for calculating the discriminant function or leave-one-out cross-validation.

	**Italy**		**United Kingdom**	

**Entire sample**				
Italy	73	91.3%	7	8.8%
United Kingdom	13	16.7%	65	83.3%

Total correctly classified 87.3%				
**Cross-validated (leave-one-out)**				
Italy	71	88.8%	9	11.3%
United Kingdom	15	19.2%	63	80.8%
Total correctly classified 84.8%				

## Discussion

With regard to the first hypothesis we made, *an additional video presentation has a positive influence on Perceived Usefulness (PU) and Intention to Use (ITU) the robot*, the result of our experiment demonstrates that, when a live demonstration and interaction session is available, a video presentation doesn’t significantly change the participant’s perception of the robot in comparison with a simple oral presentation by an expert researcher. This confirms that the physical embodiment of the robot has a huge impact on the users, especially in the first encounters, as shown in Bainbridge et al. (2011), Krogsager et al. (2014), which seem to override other information.

This result can be explained by the interrelations of the constructs found by Heerink et al. (2010). In fact, in the context of social robotics, the PU and ITU are influenced by the social interaction and perceived capabilities of the robot, which can be evaluated only through a live demonstration, otherwise these are derived from previous experience or just imagined. Therefore, when the participants have the opportunity to interact with the robot and directly experience its capabilities, this experience is likely to form their perception and influence their opinion more than a video or an oral presentation.

The second hypothesis is confirmed as, *Perceived Usefulness (PU) and Intention To Use (ITU) a robot in the future profession are different between English and Italian psychology students*. Indeed, our experiment showed a significant difference and, therefore, provided support for a positive answer. The Italian students had the most positive ITU and PU, while the English students were split in half for the PU and mainly negative for the ITU.

This confirms the strong relationship between PU and ITU, i.e., the former mainly determines the latter, which has been found for psychology students and practitioners in a previous experiment (Conti et al., 2017c). In fact, scores are very similar for all the other factors that determine the ITU according to (Heerink et al., 2010), i.e., ATT, PENJ, and SI.

Analyses of the data with the decision tree and discriminant methods confirm that positive ITU and PU are distinctive of the Italian students and highlight a role for the FC. Indeed, the majority of Italian students are also characterized by low confidence in their competence (FC) in using the robot, whereas English students are split into two main groups according to FC: a minority who believe they have the necessary competences and want to use the robot and a majority who believe they don’t have the necessary competences to make a good use of the robot, and therefore don’t want to use it.

This result highlights a cultural difference between the two groups. As pointed out by Hofstede (1980), when comparing English and Italians, the former are more pragmatic and do not want to use a novel technology that they still see at a research stage, while the latter will tend to take risks rather easily and are more open to adopting novel appliances in the workplace. These behaviours may explain the positive ITU of robots among most Italian students even if they score FC even lower than the English students.

### Limitations of This Study

“Culture” in our research means just geographical discrimination, and we did not investigate which cultural characteristics constrained individual respondents, based on specific determinants such as the ones presented in social science literature. The term “culture” in this work refers to the countries involved in this study and did not consider the full ideas or social behaviour of every participant. At the same time as this is a common tactic in cross-cultural studies, both populations were recruited from university campuses and therefore, there is a possibility that the presented student’s viewpoints are not representative for each country as a cultural whole.

The study could be limited by the relatively brief interaction between the students and the NAO robot in which they should have learned the potential applications, have updated the previous knowledge of the specific technology. On the other hand, in this work the focus was on the students’ general perception of SAR as a tool for education and care, and we did not intend to test the specific *Choregraphe* platform. The software used for programming the robot used a scripted sequence of behaviours which means no extra intelligence algorithms have been added to the robot’s performance. It is obvious that in this short time, the robot would not be capable to adapt its reactions to many situations especially when it was facing unpredictable behaviours of humans. The students may have missed learning this additional capability in the interaction with the robot. This limitation could be explored in future studies by adding examples of adaptive intelligence algorithms in a longer interaction between the students and the NAO robot. Also, they could include a questionnaire targeted to understanding how much the person is knowledgeable of what a robot can do “in general.”

## Conclusion and Future Work

Novel artificial intelligence technologies and robotics platforms are opening several new possibilities in the care of people and provide evidence in support of their use in psychological practice. It is expected that they will have the same great positive impact that computers had a few decades ago. As when computers were introduced, some studies pointed out the scepticism of practitioners and the hostile attitude of the general population, who see the robots as dangerous and unfit for the role of clinical and social assistants. However, these studies didn’t usually allow participants to interact with a real robot or experience a demonstration of the actual applications, but relied solely on their previous knowledge, which could be based on science fiction or media portrayals.

In this paper, we investigated the perception and intention to use a humanoid robot in psychology students, after a live presentation of a robotic platform with examples of its successful usage in therapeutic and educational contexts. Psychology students are candidates to see the full benefits of current research when the prototypes will be validated for clinical use and protocols will be established for making robots an important part of psychological practice like computers are now.

For data collection, we used an established questionnaire, based on the Unified Theory of Acceptance and Use of Technology (UTAUT) model, which was completed by 158 psychology students from two universities in different countries: Catania (Italy) and Plymouth (United Kingdom).

Statistical analysis showed that most of the participants had low anxiety and a positive attitude in using the robot in their profession. This positive attitude contrasts with the negative one of the general public, and the possibility to have real interaction with the robot certainly had a positive influence. However, just a few participants (16% of the overall sample) believed themselves to have the necessary abilities to make good use of it, i.e., disagreed on the facilitating conditions (FC) statements. This lack of confidence negatively influences the intention of English students to use the robot, the majority (59%) of whom disagreed to using the robot in the near future. On the contrary, responses to our questionnaire show that the majority of Italian students, who are culturally more inclined to take a risk, perceived the usefulness (PU: 73% positive) and were willing to use the robot (ITU: 63% positive) even if their confidence in having the necessary skills was even lower than that of the English students (FC: Italy 2.33 < United Kingdom 2.5).

In general, we see that both groups scored the statements related to FC very low, meaning that they felt both curricula did not provide enough knowledge or practical skills to use a robot. This is unfortunately true because robotics is seen as a very distant field from Psychology, but the authors believe that it would improve things if we could introduce robotic assistants in psychological practice. The solutions are very much related to the teaching of basic computer programming skills in psychology education, in both the United Kingdom and Italy. Therefore, future research might consider exploring the extent of the programming skills that would facilitate the use socially assistive robotics by future psychologists. Future studies should investigate more in deep the reasons behind the student’s intention to use or not the robotic technology as part of their planned career.

## Data Availability

The datasets generated during and/or analyzed during the current study are available from the corresponding author on reasonable request.

## Ethics Statement

This study was carried out in accordance with the recommendations of the relevant University boards in Plymouth and Catania with written informed consent from all subjects. All subjects gave written informed consent in accordance with the Declaration of Helsinki. The protocol was approved by the relevant ethical boards of the University of Plymouth and University of Catania.

## Author Contributions

DC, AC, and AD curated the data, formally analyzed the data, provided the methodology, validated the data, and wrote the original draft of the manuscript. DC and AD investigated the data and provided the resources. AD administrated the project. AC, SD, and AD supervised the study. DC visualized the data. All authors wrote, reviewed, and edited the manuscript.

## Conflict of Interest Statement

The authors declare that the research was conducted in the absence of any commercial or financial relationships that could be construed as a potential conflict of interest.

## Appendix

**TABLE:** Questionnaires: Constructs and number of items.

**Construct**	**No. of items**	**Code**	**Questions (English) (Heerink et al., 2010)**	**Questions (Italian)**
ANXiety	4	ANX	(1) I should use the robot, I would be afraid to make mistakes with it.	(1) Se dovessi usare il robot, avrei paura di commettere errori.
			(2) If I should use the robot, I would be afraid to break something.	(2) Se dovessi usare un robot, avrei paura di rompere qualcosa.
			(3) I find the robot scary.	(3) Io trovo che il robot incute timore.
			(4) I find the robot intimidating.	(4) Trovo che il robot mi intimidisce.
ATTitude	3	ATT	(5) I think it’s a good idea to use the robot.	(5) Penso che sia una buona idea usare il robot.
			(6) The robot would make my future job more interesting.	(6) Il robot renderebbe il mio futuro lavoro più interessante.
			(7) It’s good to make use of the robot.	(7) È bene fare uso del robot.
Facilitating Conditions	2	FC	(8) I have everything I need to make good use of the robot.	(8) Ho a disposizione tutto quanto mi serve per usare al meglio un robot.
			(9) I know enough of the robot to make good use of it.	(9) Ne so abbastanza del robot per farne un buon uso.
Intention To Use	3	ITU	(10) I think I’ll use the robot in the near future.	(10) Penso che userò il robot in un futuro prossimo.
			(11) I am certain to use the robot in the near future.	(11) Certamente userò il robot in un futuro prossimo.
			(12) I’m planning to use the robot in the near future.	(12) Sto pensando di usare il robot in un futuro prossimo.
Perceived ADaptability	3	PAD	(13) I think the robot can be adaptive to what I need.	(13) Penso che il robot può fare ciò che mi serve.
			(14) I think the robot will only do what I need at that particular moment.	(14) Penso che il robot farà solo ciò che mi serve in quel particolare momento.
			(15) I think the robot will help me when I consider it to be necessary.	(15) Penso che il robot mi aiuterà quando lo riterrò necessario.
Perceived ENJoyment	5	PENJ	(16) I enjoy the robot talking to me.	(16) Mi piace che il robot parli con me.
			(17) I enjoy doing things with the robot.	(17) Mi piace fare attività con il robot.
			(18) I find the robot enjoyable.	(18) Trovo che il robot sia piacevole da usare.
			(19) I find the robot fascinating.	(19) Trovo che il robot sia esteticamente piacevole.
			(20) I find the robot boring.	(20) Trovo che il robot sia noioso.
Perceived Sociability	4	PS	(21) I consider the robot a pleasant conversational partner.	(21) Ritengo che il robot sia un piacevole interlocutore.
			(22) I find the robot pleasant to interact with.	(22) Trovo che sia piacevole interagire con il robot.
			(23) I feel the robot understands me.	(23) Sento che il robot mi capisce.
			(24) I think the robot is nice.	(24) Penso che il robot è gentile.
Perceived Usefulness	3	PU	(25) I think the robot is useful to me.	(25) Penso che il robot mi sia utile.
			(26) It would be convenient for me to have the robot.	(26) Sarebbe comodo per me avere il robot.
			(27) I think the robot can help me with many things.	(27) Penso che il robot mi può aiutare in molte cose.
Social Influence	2	SI	(28) I think the staff would like me using the robot.	(28) Penso che al personale piacerebbe che io usassi il robot.
			(29) I think it would give a good impression if I should use the robot.	(29) Penso che darei una buona impressione se dovessi usare il robot.
Social Presence	5	SP	(30) When interacting with the robot I felt like I’m talking to a real person.	(30) Quando ho interagito con il robot ho sentito come se stessi parlando con una persona reale.
			(31) It sometimes felt as if the robot was really looking at me.	(31) A volte ho avuto l’impressione come se il robot mi stesse davvero guardando.
			(32) I can imagine the robot to be a living creature.	(32) Io posso immaginare il robot come una creatura vivente.
			(33) I often think the robot is not a real person.	(33) Ho pensato spesso che il robot non-sia una persona reale.
			(34) Sometimes the robot seems to have real feelings.	(34) A volte il robot mi sembra provare vere sensazioni.
TRUst	2	TRU	(35) I would trust the robot if it gave me advice.	(35) Mi fiderei del robot se questo mi desse un consiglio.
			(36) I would follow the advice the robot gives me.	(36) Vorrei seguire il consiglio che il robot mi dà.
