# Supplementary material for: Are Future Psychologists Willing to Accept and Use a Humanoid Robot in Their Practice? Italian and English Students’ Perspective
Source: Front Psychol. 2019 Sep 18;10:2138. doi: 10.3389/fpsyg.2019.02138 (PMC6759609; doi:10.3389/fpsyg.2019.02138)
Supplement: Supplementary file 1 [file Table_1.DOCX]

# Supplementary Material

Script followed by the presenter

“Hello everyone and thank you for participating in our research. Thank you for signing the informed consent and I would remind you that you can leave the room at any time, without having to give any further explanations.

The robot you see here is NAO, which is a small toy-like humanoid robot manufactured by Softbank Robotics. Thousands of these robots were sold worldwide and their safety is guaranteed by design and extensive tests. NAO is 58 cm high; weights 4.3 kg and it can be programmed to produce gestures with its arms, hands, legs, head, and body. NAO can detect faces and mimic eye contact by moving its head accordingly, it can also vary the colour of LEDs in its eyes contours to simulate emotions, and it can capture a lot of information about the environment using sensors and microphones. Nao is programmed with a graphical programming tool, named *Choregraphe* that you can see projected behind me. An example is shown in the laptop (Figure 3).”


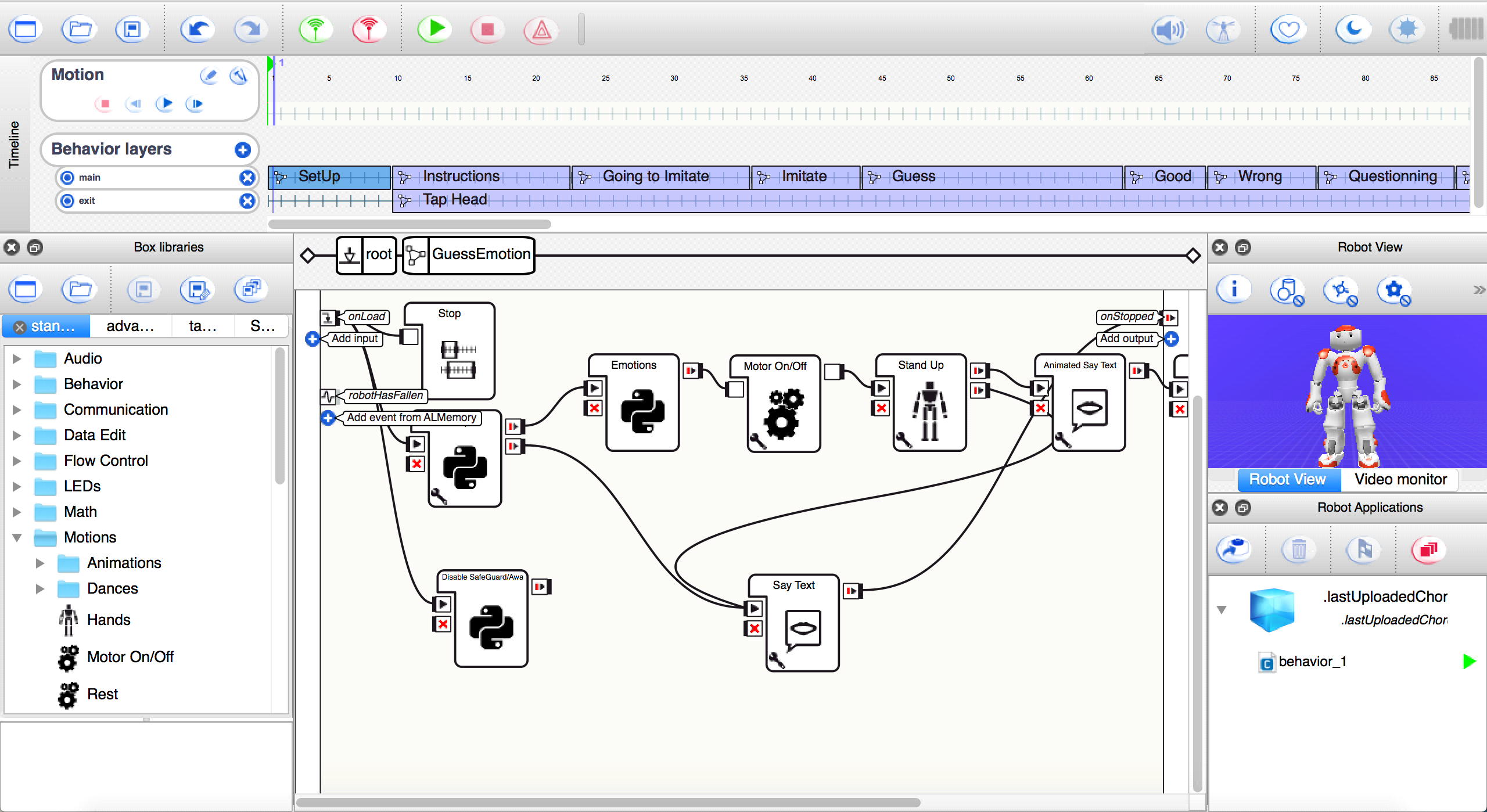


**Figure 3** *Choregraphe* platform example

“This programming tool provides an intuitive way to design complex behaviours by combining pre-programmed blocks.

You will be able to freely interact with the robot and ask me questions shortly, but first, let me give some examples of the several research projects in which NAO has been successfully used. Amongst the many applications, we have selected four examples of situations very close to education and clinical psychology.

For instance, NAO was tested as a storyteller in a primary school. The aim of the study was to understand the influence of different social behaviour on over 80 preschool children’s perception of stories narrated either by NAO humanoid robot or by a human teacher. Four conditions were considered: static human, static robot, expressive human and expressive robot. Two stories, with knowledge and emotional content, were narrated in two different encounters. After each story, children were asked to draw what they recalled of the story. Analysis of the drawings shows a positive effect in recalling story details of the expressive behaviour in robot storytelling, whose efficiency is comparable to the human with the same behaviour or better if the expressive robot is compared with a static inexpressive human.

NAO could help therapists in the training imitation skills of children with Autism Spectrum Disorders. This was shown also in the case of comorbidity with Intellectual Disability, which, as you know, is the most difficult case for a clinical psychologist. The research integrated NAO in the standard therapy and the results confirmed that the robot can engage autistic children in an imitation game in which, first, the child learns to imitate the robot’s movements, then the robot imitates the child. A follow-up assessment demonstrated that the children can generalise to human-human interaction and maintain these skills after three months.

Another research study shows that NAO could help children to reduce their distress during their annual flu vaccination. This was a randomized controlled study in which children were randomly assigned to a vaccination session with a nurse who used standard administration procedures, or with a robot who was programmed to use cognitive-behavioural strategies to help them to relax while a nurse administered the vaccination. Measures of pain and distress were completed by children, parents, nurses, and researchers. It was found that children who interacted with NAO during their vaccination experienced less pain and distress than children who did not have this interaction.

Finally, NAO was introduced in hospital settings to help adults, especially the elderly, to avoid complications such as cognitive decline and depression. NAO humanoid robot conversed, made jokes, played music, danced and exercised with 49 patients aged between 18–100 in a hospital, 7 of whom had dementia. The results of the study show that most of the patients enjoyed their interaction with NAO, therefore reducing their loneliness and helping them to be mentally engaged.

Now, we will give you a questionnaire. I would remark that this research aims to find out your views as future psychologists, about the use of humanoid robots in your professional future.

The answers will be anonymous, we just need some generic details: gender, age, and nationality. You can indicate your level of agreement with the 36 statements on five-point Likert scales: 1 if you "totally disagree" with a statement, 2 if you "disagree", 3 if "neither agree nor disagree”, 4 indicates that you "agree", and 5 you will "totally agree" with the statement. We ask you to circle or cross the most suitable answer for you.

There are no right or wrong answers, but only your opinion that will remain anonymous.

Thanks again for your participation.”

| Video presentation  For the description of the activity in each segment of the see the script used by the presenter | | | | |
| --- | --- | --- | --- | --- |
| **Time** | **Duration** | **Segment name** | | **Example pictures** |
| 00:00  01:49 | 1.49' | Storytelling in a primary school  (Conti, Cirasa, Di Nuovo, & Di Nuovo, 2019) | | 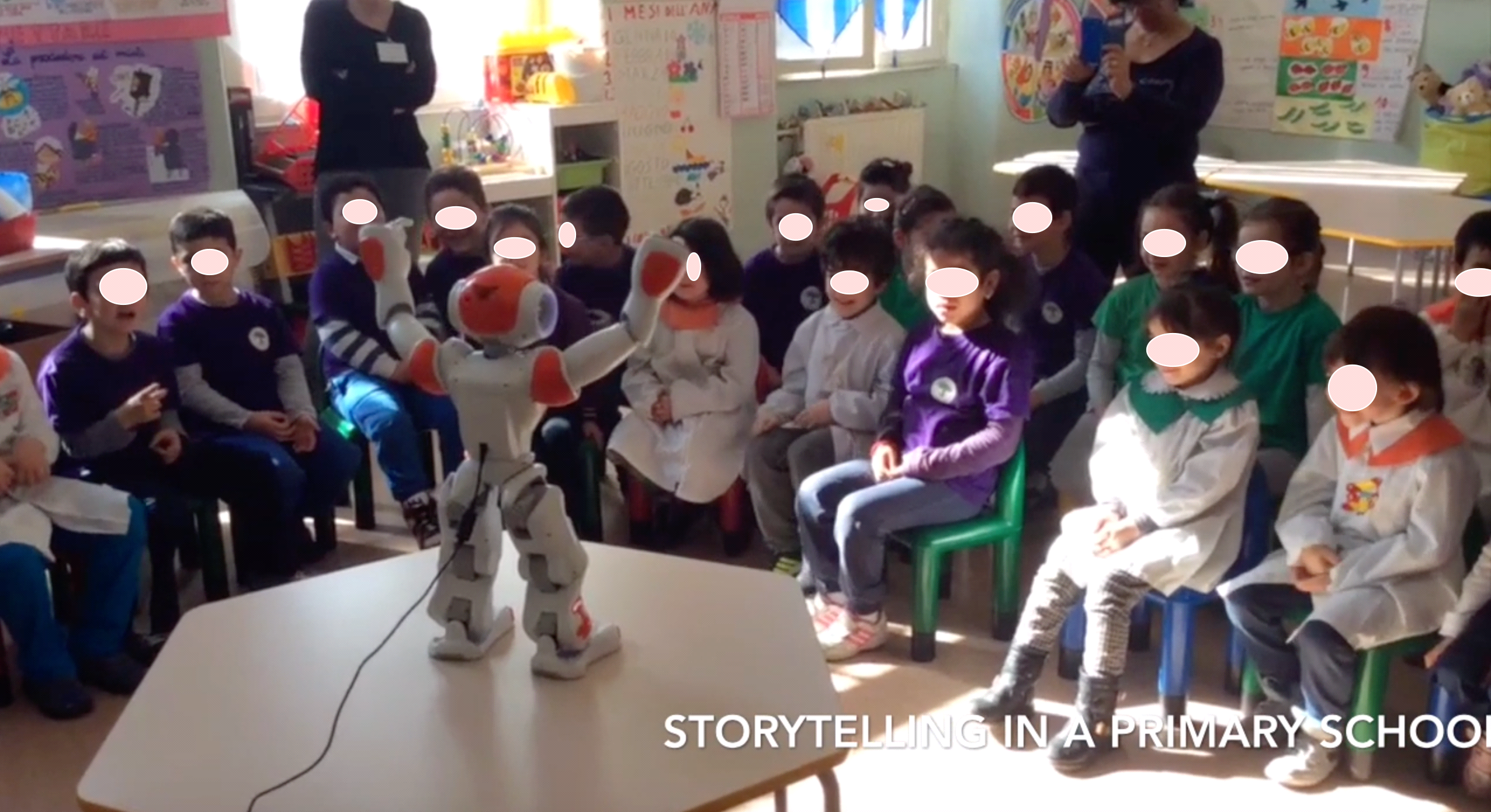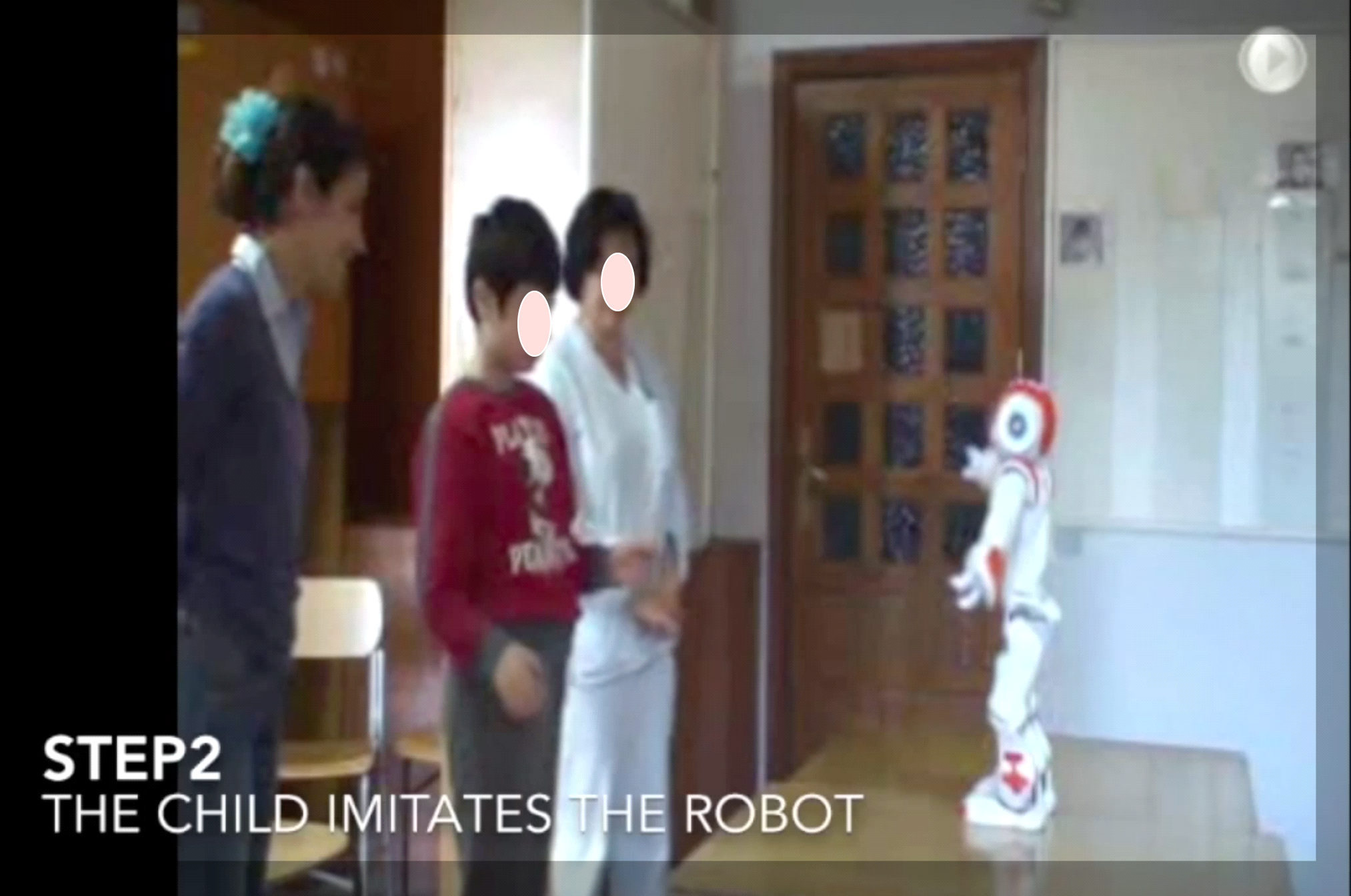 |
| 01:50  03:04 | 1.54' | Children with Autism Spectrum Disorders  (Conti, Di Nuovo, Buono, Trubia, & Di Nuovo, 2015) | |  |
| 03:05  04:55 | 1.50' | … in a hospital with Children (Beran, Ramirez-Serrano, Vanderkooi, & Kuhn, 2013)  [www.youtube.com/watch?v=Y4EFjEX6o90](http://www.youtube.com/watch?v=Y4EFjEX6o90) | | 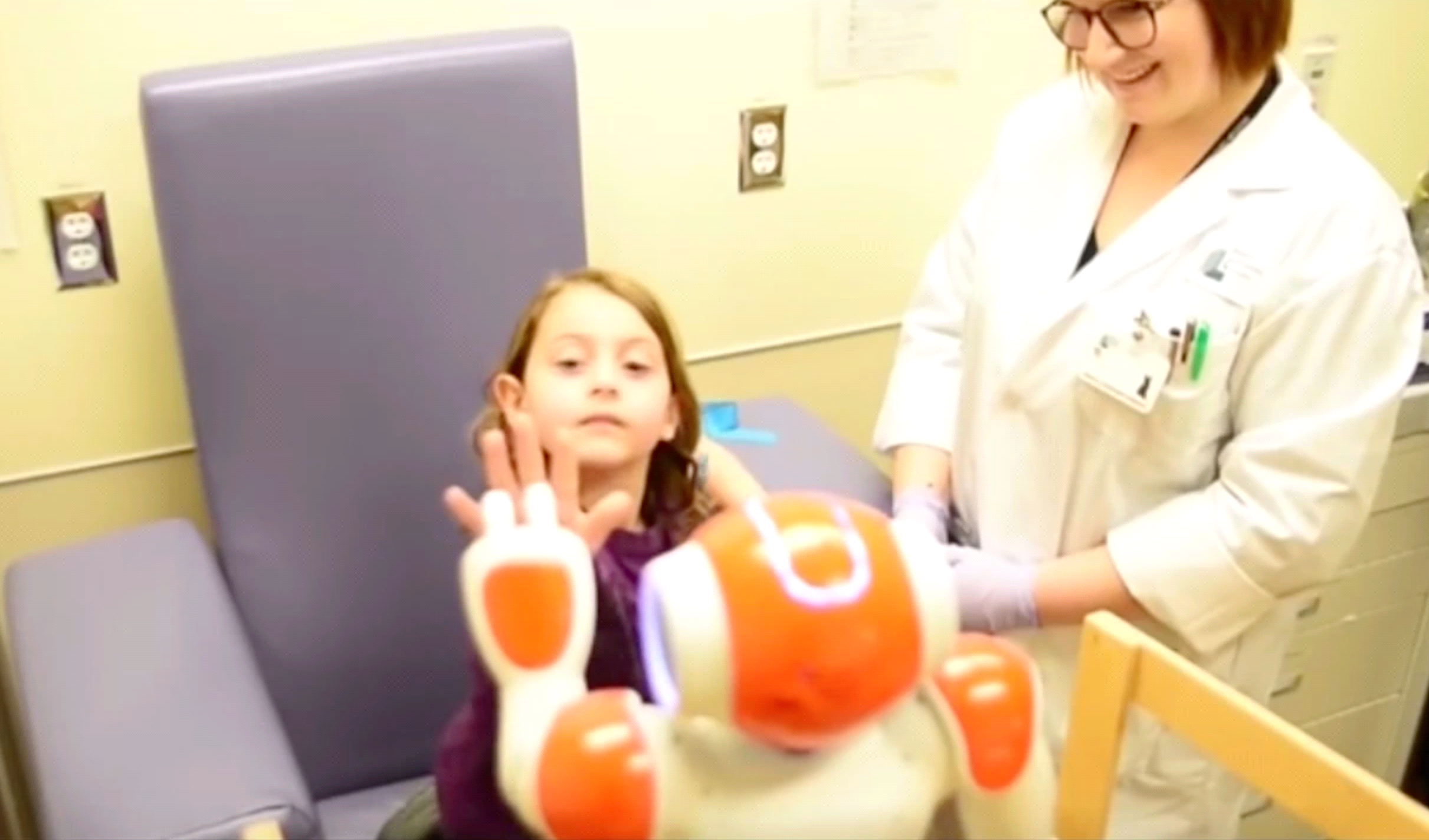 |
| 04:56  06:11 | 1.55' | … and in hospital with Adults (Sarabia et al., 2018)  [www.youtube.com/watch?v=xhEsXBV7dFI](http://www.youtube.com/watch?v=xhEsXBV7dFI) | 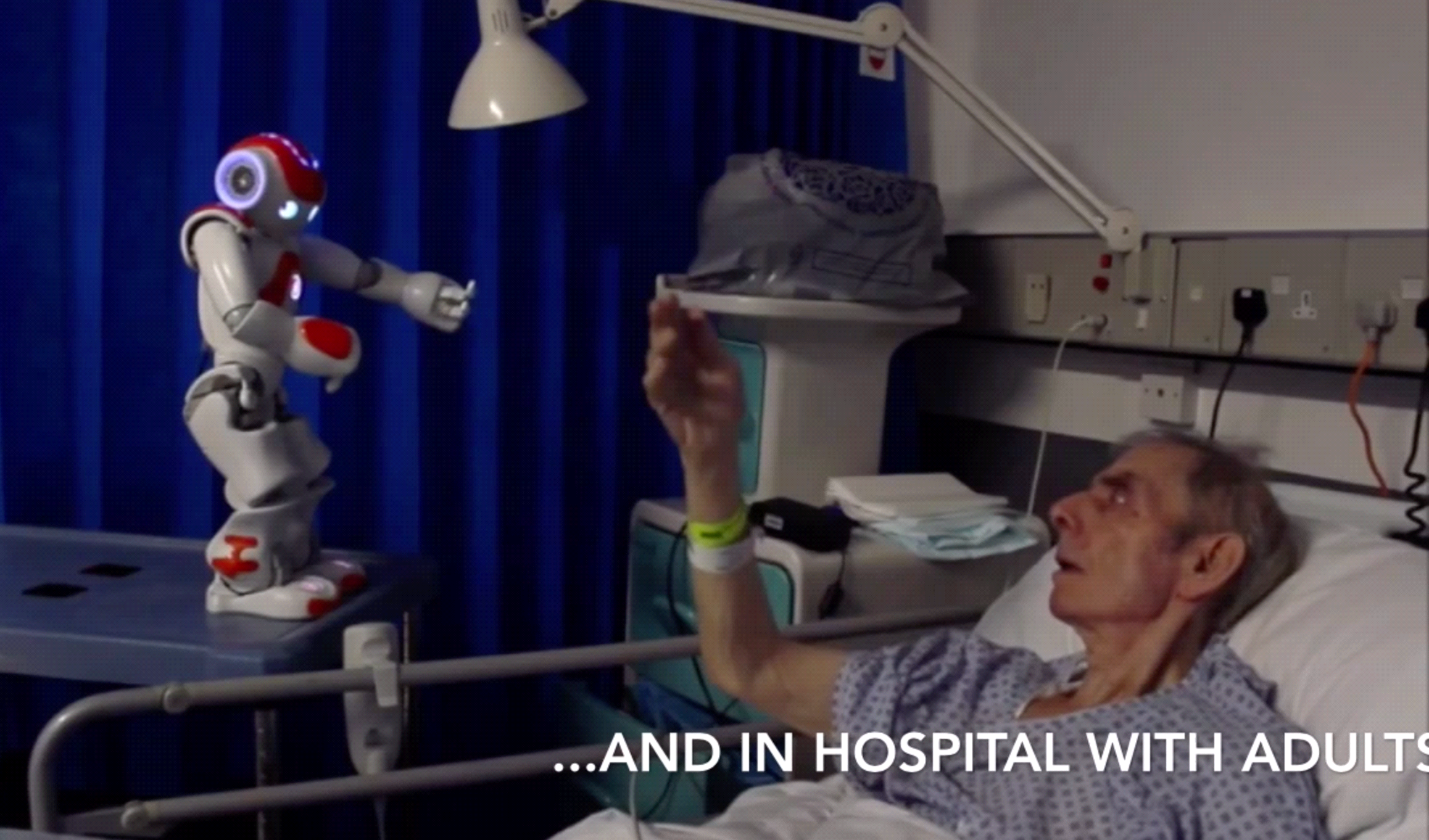 | |

Sequence of activities during the live NAO robot interaction session

1. The NAO robot performs welcoming and greeting actions.
2. The robot dances to show agility and movement capability (1 minute)
3. The robot answers to students’ questions (10 minutes). The presenter should facilitate by giving some examples of possible questions e.g. Can you look at me?, What’s your name?, Who made you?, Can you understand me? etc,.
   Students receive a list of all possible questions and, in turn, speaks to the robot, testing its speech recognition abilities. Topics were varied: (for full list you can see <http://doc.aldebaran.com/2-1/nao/basic_channel_conversation.html>)
4. The NAO robot proposes an interactive game of image recognition (3 minutes). Sheets with printed images is place on the table that a random volunteer selected and show to the robot when ask questions such as “Show me a tree” or “I would like to see a star”. The game is repeated 3 times with different volunteers (total 9 minutes).
5. The NAO identify and follow a red ball. Volunteers move the ball (max 1 minute each, max 3 minutes).
6. The NAO asks to place in its hand an object. It then grasps the object, but return it saying that the object is not interesting.
7. The robot is place on the floor in a walking mode (5 minutes). The robot walks around the room among the participants according to commands given by volunteers (directions: forward, backward, turn left and right)
8. The robot thanks the participants for attending and for their participation.
